# Case report of a spontaneous pneumothorax after the recovery from COVID‐19 pneumonia: A delayed complication

**DOI:** 10.1002/ccr3.4971

**Published:** 2021-10-18

**Authors:** Sangam Shah, Apil Pokhrel, Rajan Chamlagain, Yagya Raj Adhikari, Bipin Kandel, Roman Dhital, Basanta Sharma Paudel, Sutap Yadav

**Affiliations:** ^1^ Maharajgunj Medical Campus Institute of Medicine Tribhuvan University Maharajgunj Nepal; ^2^ Tribhuvan University Teaching Hospital Maharajgunj Nepal; ^3^ Department of Cardiology Institute of Medicine Tribhuvan University Maharajgunj Nepal; ^4^ Department of Cardiology Manmohan Cardio Thoracic Vascular and Transplant Canter Maharajgunj Nepal

**Keywords:** complication, COVID‐19, pneumothorax

## Abstract

This case demonstrates pneumothorax as a consequence of COVID‐19 and emphasizes the significance of follow‐up of the COVID‐19 patients.

## Graphical abstract

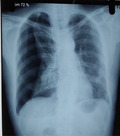

## 1 INTRODUCTION

Acute respiratory distress syndrome, disseminated intravascular coagulation, and cytokine storm are consequences of COVID‐19. Pneumothorax is an unusual complication of COVID‐19 that further worsens the hypoxia and requires immediate intervention. We present a case of a 30‐year‐old male with pneumothorax without underlying risk factors for pneumothorax apart from a recent COVID‐19 infection.

COVID‐19, which first appeared in China's Wuhan city in December, has been labeled a global pandemic by the World Health Organization (WHO). COVID‐19 causes severe pneumonia which is caused by enveloped RNA virus (SARS‐CoV‐19). Acute respiratory distress syndrome (ARDS), disseminated intravascular coagulation (DIC), and cytokine storm are all consequences of COVID‐19.^1^, ^2^, ^3^ COVID‐19 was previously considered to primarily cause pulmonary parenchymal damage; however, extra‐parenchymal symptoms including pulmonary thromboembolism, pleural effusion, empyema, and pneumothorax were revealed later.^4^ Secondary spontaneous pneumothorax (SP) caused by COVID‐19 has been recorded on several occasions at the time of diagnosis or during therapy. However, late‐onset spontaneous pneumothorax in individuals who recovered from COVID‐19 is rare.^5^ Here, we present a case of an adult male with previously diagnosed COVID‐19 who developed spontaneous pneumothorax after seventeen days of recovery.

## 2 CASE REPORT

A 30‐year‐old male presented to the emergency department of our hospital with the chief complaints of shortness of breath and burning sensation of chest for five days. The shortness of breath was gradual in onset, occurred even at rest, and was increasing. The burning sensation of chest was associated with cough. The patient had a history of 7 days of hospitalization due to bilateral COVID‐19 pneumonia, seventeen days before the recent presentation. He had no history of fever, bilateral pedal edema, palpitation, and had no known comorbidities. He did not smoke or consumed alcohol. He was a serviceman with no occupational exposure to any known lung irritants.

He was ill‐looking, not oriented to time, place, and person, and Glasgow Coma Scale (GCS) was 13/15. His body temperature was 98°F, oxygen saturation was 96% in room air, blood pressure was 160/110 mm Hg, pulse rate was 128 beats/min that was regular with normal volume, and respiratory rate was 24 breaths/min. He had no pallor, icterus, clubbing, cyanosis, and edema on general physical examination. Chest examination revealed decreased air entry on the right side of chest.

**FIGURE 1:**
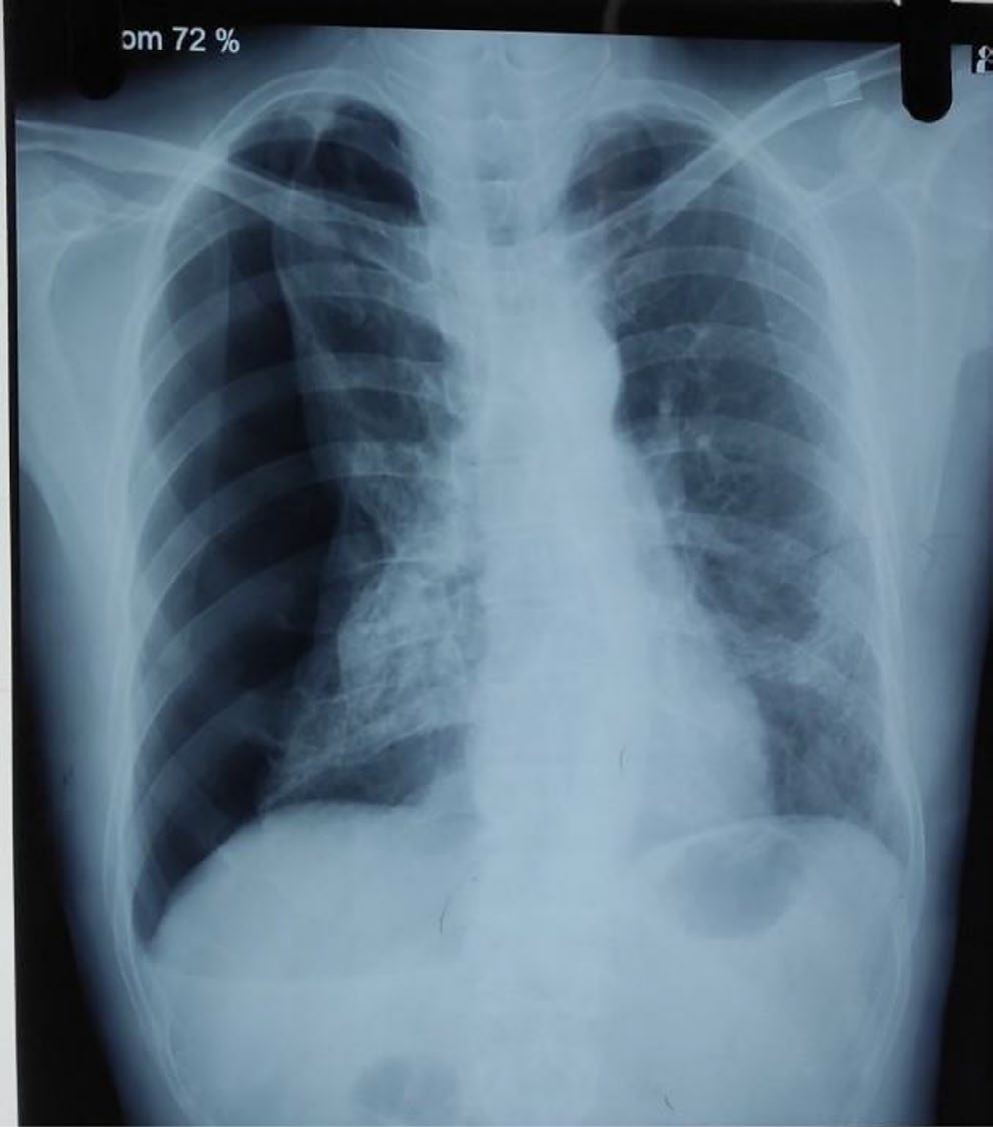
Chest X‐ray showing right‐sided pneumothorax

Laboratory examination at the time of admission revealed hemoglobin 12.9 gm % and hematocrit 44.2% suggesting no polycythemia. His total leukocyte count was 16,190 cells/mm^3^, neutrophil 88%, lymphocyte 8%, and platelet count 472,000 cells/mm^3^. The random blood sugar level was 152 mg/dl, D‐dimer was 350 (0–230 ng/ml), and fibrinogen was 510 (270–500 mg/dl). Chest X‐ray PA view revealed visceral pleural edge making a sharp line with no vascular markings in the right side suggesting right‐sided pneumothorax (Figure 1).

He was diagnosed with post‐COVID‐19 right‐sided pneumothorax. Following this, 28F chest tube was inserted on the right fifth intercostal space at the mid‐axillary line under aseptic conditions, and gush of air was noted after the pleural puncture. Intravenous (IV) antibiotics (amoxicillin and clavulanic acid) and oral analgesics (paracetamol and tramadol) were given after the procedure. The chest tube was removed after 6 days when his vitals were stable. He was discharged on the 9th day of admission with oral medications (amoxicillin and clavulanic acid [500 mg + 125 mg] TDS, and tapering dose of prednisolone [40 mg]). The patient had no complications on follow‐up after one month.

## 3 DISCUSSION

The cases of spontaneous pneumothorax have been reported in SARS‐COVID‐19 pneumonia patients. The presence of air within the chest cavity is known as pneumothorax, and it can be classified as either spontaneous or traumatic pneumothorax.^2^ Traumatic pneumothorax can result from any form of injury, including iatrogenic injuries like those caused by central line placement. Primary and secondary pneumothorax are the two types of spontaneous pneumothorax. A primary pneumothorax develops when there is no obvious underlying chest or lung disease.

The symptoms of COVID‐19 pneumonia range from mild to severe. The development of pneumothorax after the complete recovery of COVID‐19 pneumonia is rare. The exact pathogenesis of pneumothorax in patients with COVID‐19 has not been fully understood because only 1% of all hospitalized COVID‐19 patients develop this complication and sufficient studies are lacking.^6^ Although the etiology of COVID‐19 pneumonia pneumothorax is unknown, our case report shows a causal relationship between the two diseases.

Preexisting pulmonary cysts or pneumatoceles have been reported to be a contributing factor in such patients, particularly those who require positive pressure ventilation, according to the literature.^7^, ^8^ Although we did not have a prior CT chest imaging in our case to rule out the possibility of a preexisting cyst or pleural blebs, our patient's history revealed no indication or suspicion of such problems. Furthermore, positive pressure ventilation was not required for our patient. The second reason would be increase in intrathoracic pressure caused by a continuous cough, which causes peripheral‐irritated alveoli to rupture into the pleural cavity, resulting in pneumothorax.^9^, ^10^

Pulmonary embolism with subsequent pulmonary infarction and cavity formation can also be a contributing cause when the cavity ruptures into the pleural space.^11^ There was no clinical or radiologic evidence of pulmonary embolism or infarction in our patient. Our patient developed pneumothorax after apparent recovery from COVID‐19 pneumonia without any known triggering event or trauma. The Maclin effect, which occurs when the intra‐alveolar pressure rises, might have caused the alveolar wall to rupture.^12^

The pneumothorax with spontaneous or barotraumatic cause is treated with a chest tube drainage. Chest tube placement will be more difficult in mechanically ventilated patients because positive pressure ventilation can contribute to extended air leak due to barotrauma. Surgical intervention may require if there is persistent air leak despite chest tube insertion.

To limit and reduce the risk of infection transmission, many cardiovascular and thoracic surgery organizations advocate certain preventative procedures for chest tube drainage installation in COVID‐19 patients.^13^ The treatment is based on the etiology. Traditional water seal chest drain systems can be utilized safely by adding bleach to the water seal chamber and employing an inline virus filter between the chest drain system and the wall suction system, according to the American Association for Surgery of Trauma.^13^, ^14^ Malpositioning, repeated pneumothorax, infection (including empyema), organ or artery injury, and reexpansion pulmonary edema are common problems of chest tube implantation.

## 4 CONCLUSION

With the rapid rise of COVID‐19‐infected patients worldwide, it is important to understand complications of this illness and respond promptly. This case demonstrates pneumothorax as a consequence of COVID‐19 and emphasizes the significance of follow‐up of the COVID‐19 patients.

## CONFLICT OF INTEREST

None.

## AUTHOR CONTRIBUTIONS

SS conceptualized the study and reviewed and edited the manuscript, and was in charge of the case. SS, RC, and AP wrote the original manuscript and reviewed and edited the manuscript. SS, RC, AP, BK, YRA, RD, BSP, and SY were in charge of the case and reviewed and edited the manuscript.

## ETHICAL APPROVAL

Written informed consent was obtained from the patient for publication of the case report.

## CONSENT

Written informed consent was obtained from the patient.

## Data Availability

All the required information is available in the manuscript itself.

## References

[1] Douedi S , Miskoff J . Novel coronavirus 2019 (COVID‐19): a case report and review of treatments. Medicine. 2020;99(19):e20207. 32384516 10.1097/MD.0000000000020207 PMC7220032

[2] Douedi S , Chaudhri M , Miskoff J . Anti‐interleukin‐6 monoclonal antibody for cytokine storm in COVID‐19. Ann Thorac Med. 2020;15(3):171. 32831940 10.4103/atm.ATM_286_20 PMC7423207

[3] Singh A , Bass J , Lindner DH . Rare complication of pneumomediastinum and pneumopericardium in a patient with COVID‐19 pneumonia. Case Rep Pulmonol. 2020;2020:1‐4. 10.1155/2020/8845256 PMC7652632 33204564

[4] Kasturi S , Muthirevula A , Chinthareddy RR , Lingaraju VC . Delayed recurrent spontaneous pneumothorax post‐recovery from COVID‐19 infection. Indian J Thorac Cardiovasc Surg. 2021;37(5):551‐553. 10.1007/s12055-021-01145-w PMC7851320 33551586

[5] Sayan M , Turk MS , Ozkan D , Kankoc A , Tombul I , Celik A . Hemopneumothorax as an unusual and delayed complication of coronavirus disease 2019 pneumonia. J Chest Surg. 2021;10‐2. 10–12. 10.5090/jcs.20.149 PMC8646073 33767017

[6] Sahagun J , Chopra A , David AG , Dao D , Chittivelu S . Secondary spontaneous pneumothorax in a COVID‐19 recovered patient case presentation. Cureus. 2021;13(7):1‐5. 10.7759/cureus.16415 PMC8364669 34401214

[7] Martinelli AW , Ingle T , Newman J , et al. COVID‐19 and pneumothorax: a multicentre retrospective case series. Eur Respir J. 2020;56(5):2002697. 32907891 10.1183/13993003.02697-2020 PMC7487269

[8] Zhou C , Gao C , Xie Y , Xu M . COVID‐19 with spontaneous pneumomediastinum. Lancet Infect Dis. 2020;20(4):510. 32164830 10.1016/S1473-3099(20)30156-0 PMC7128610

[9] Sun R , Liu H , Wang X . Mediastinal emphysema, giant bulla, and pneumothorax developed during the course of COVID‐19 pneumonia. Korean J Radiol. 2020;21(5):541. 10.3348/kjr.2020.0180 32207255 PMC7183834

[10] Joynt GM , Antonio GE , Lam P , et al. Late‐stage adult respiratory distress syndrome caused by severe acute respiratory syndrome: abnormal findings at thin‐section CT. Radiology. 2004;230(2):339‐346. 10.1148/radiol.2303030894 14752179

[11] Bompard F , Monnier H , Saab I , et al. Pulmonary embolism in patients with COVID‐19 pneumonia. Eur Respir J. 2020;56(1):2001365. 32398297 10.1183/13993003.01365-2020 PMC7236820

[12] Abushahin A , Degliuomini J , Aronow WS , Newman TG . A case of spontaneous pneumothorax 21 days after diagnosis of coronavirus disease 2019 (COVID‐19) pneumonia. Am J Case Rep. 2020;21:1‐4. 10.12659/AJCR.925787 PMC7447295 32798215

[13] Sihoe A , Filosso P , Cusumano G , Lococo F , Melfi F . Pneumomediastinum and pneumothorax in COVID‐19 patients [1]. 2021. p. 1–8.

[14] Pieracci FM , Burlew CC , Spain D , et al. Tube thoracostomy during the COVID‐19 pandemic: guidance and recommendations from the AAST Acute Care Surgery and Critical Care Committees. Trauma Surg Acute Care Open. 2020;5(1):e000498. 32411822 10.1136/tsaco-2020-000498 PMC7213907

